# Leisure screen time, cardiometabolic pathways, and frailty: A two-step Mendelian randomization analysis

**DOI:** 10.1097/MD.0000000000049560

**Published:** 2026-07-03

**Authors:** Ye Cao, Qunxiong Fan, Nan Xia, Qing Hao, Yi Feng

**Affiliations:** a Department of Cardiology, Renmin Hospital, Hubei University of Medicine, Shiyan, Hubei, People’s Republic of China; b Hubei Clinical Medical Research Center for Atherosclerotic Cardiovascular Diseases, Shiyan, Hubei, People’s Republic of China; c Shiyan Key Laboratory for Atherosclerotic Disease Research, Renmin Hospital, Hubei University of Medicine, Shiyan, Hubei, People’s Republic of China; d Department of Pulmonary and Critical Care Medicine, Renmin Hospital, Hubei University of Medicine, Shiyan, Hubei, People’s Republic of China.

**Keywords:** cardiometabolic risk, frailty, leisure screen time, mediation analysis, Mendelian randomization

## Abstract

Whether leisure screen time (LST) is causally related to frailty remains uncertain. We used Mendelian randomization (MR) to examine the association of genetically proxied LST with frailty risk and to explore potential mediation through cardiometabolic factors. Summary-level genome-wide association study data for LST, frailty, and candidate mediators were obtained from large consortia. Two–sample MR was conducted using inverse–variance weighted (IVW) as the primary estimator, with MR–Egger, weighted median, weighted mode, MR-PRESSO, and radial MR used as complementary analyses. A two-step MR framework was used to assess mediation through body mass index (BMI), hypertension (HTN), coronary artery disease (CAD), and type 2 diabetes (T2D). Higher genetically predicted LST was associated with a higher risk of frailty after radial MR outlier correction (IVW OR = 1.157, 95% CI: 1.118–1.197, *P* = 5.42e^−17^), with broadly consistent directions across sensitivity estimators. Although heterogeneity was observed in the initial analysis, residual heterogeneity and directional pleiotropy were not evident after radial MR correction. In two-step MR analyses, BMI, HTN, CAD, and T2D showed separate, modest mediation signals, accounting for 11.4%, 12.7%, 7.2%, and 7.8% of the total effect, respectively. Genetic evidence was consistent with a potential causal association between higher LST and increased frailty risk. BMI, HTN, CAD, and T2D may each partly mediate this association, although the mediation estimates were modest, pathway-specific, and non-additive. Further studies with refined exposure phenotyping and interventional designs are needed to determine whether reducing screen-based sedentary behavior can help prevent frailty.

## 1. Introduction

Frailty is a geriatric syndrome characterized by reduced physiological reserve and diminished resilience to stressors, and it is increasingly recognized as a key determinant of adverse outcomes in older adults.^[1,2]^ Frail individuals face higher risks of falls, disability, hospitalization, and mortality, creating a substantial and growing burden on healthcare systems worldwide.^[3]^ In the context of population aging, identifying modifiable risk factors for frailty is therefore a public-health priority.^[4]^ Among candidate determinants, lifestyle behaviors – particularly those reflecting insufficient physical activity – have attracted considerable attention.^[5,6]^

Leisure screen time (LST), a prevalent sedentary behavior, refers to time spent watching television, using computers, or engaging with other digital devices for entertainment. As LST has become embedded in daily life, epidemiological studies have linked prolonged LST with obesity, cardiovascular disease, and type 2 diabetes.^[7–9]^ Sedentary behavior has also been implicated in the development and progression of frailty.^[10]^ However, observational estimates are vulnerable to confounding and reverse causation: functional limitations may lead frail individuals to spend more time sedentary, obscuring the direction and magnitude of any causal effect.

Mendelian randomization (MR) can help address these limitations by using germline genetic variants associated with an exposure, here LST, as instrumental variables to evaluate potential causal effects on an outcome, in this case frailty.^[11–13]^ Because alleles are randomly allocated at conception and remain fixed throughout life, MR can reduce, although not eliminate, confounding and reverse causation in observational epidemiology.

We investigated the causal effect of LST on frailty using a two-sample MR design. Given the established links between LST and cardiometabolic conditions – obesity,^[14]^ hypertension,^[15]^ coronary artery disease (CAD),^[16]^ and type 2 diabetes^[17]^ – we further conducted a two-step MR mediation analysis to evaluate their potential roles as mediators. To enhance robustness and address pleiotropy and heterogeneity, we applied multiple MR estimators and sensitivity procedures, including inverse-variance weighted (IVW) as the primary model, MR-Egger, Mendelian Randomization Pleiotropy RESidual Sum and Outlier (MR-PRESSO), and radial MR.

This study, therefore, aimed to assess genetic evidence for an association between LST and frailty and to examine whether cardiometabolic traits may act as intermediate pathways. The findings may inform future observational, mechanistic, and interventional studies of screen-based sedentary behavior and frailty, while recognizing the inferential limits of MR.

## 2. Materials and methods

### 2.1. Study design

This study adhered to the Strengthening the Reporting of Observational Studies in Epidemiology using MR guidelines.^[18]^ MR inference relies on 3 core assumptions: relevance – genetic instruments are robustly associated with the exposure (LST); independence – instruments are not associated with confounders of the exposure–outcome relationship; and exclusion restriction – instruments influence the outcome (frailty) only through the exposure. These assumptions were considered according to established MR methodological guidance.^[11–13]^ Recent cardiovascular MR applications have adopted similar instrumental-variable assumptions when using genome–wide association study (GWAS) summary statistics.^[19]^

**Figure 1.**
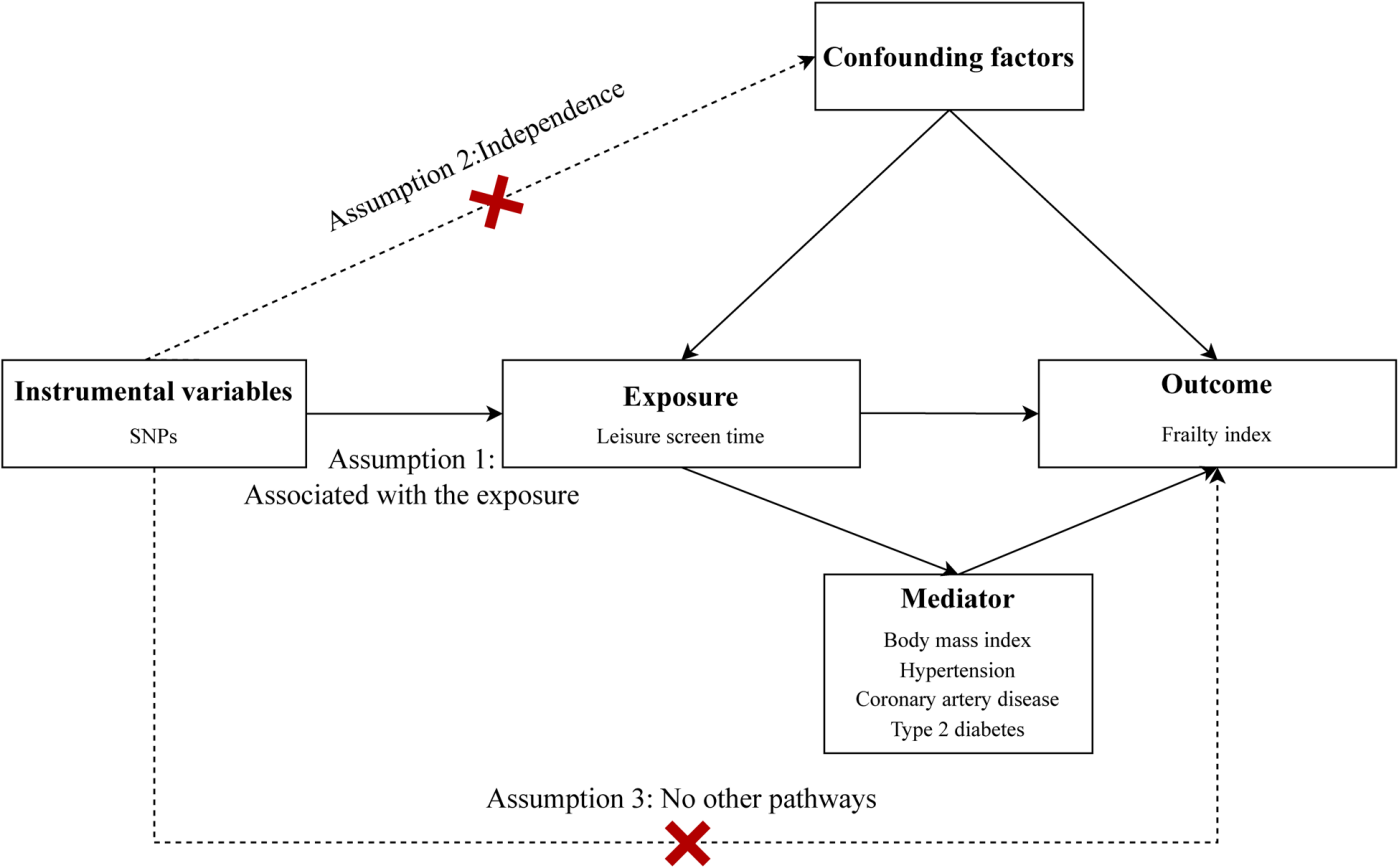
Flowchart of this MR study. MR = Mendelian randomization.

We implemented a two-sample MR framework to estimate the causal effect of genetically proxied LST on frailty (Fig. 1). To explore potential pathways, we further conducted a two-step MR analysis evaluating body mass index (BMI), hypertension (HTN), CAD, and type 2 diabetes (T2D) as candidate mediators. Analytical details and sensitivity procedures are described in subsequent sections.

### 2.2. Data sources

Summary statistics for LST were obtained from a GWAS meta-analysis of 51 cohorts (GWAS ID: GCST90104339; n = 526,725).^[20]^ LST, which included television viewing, video gaming, and computer use, was analyzed as a continuous trait (mean 3.53 hours/day; standard deviation 1.87). The exposure was assessed using domain-specific self-report items capturing the duration and intensity of screen-based leisure activities. Thus, LST should be interpreted as a heterogeneous, self-reported screen-based behavior rather than as a single uniform exposure.

Frailty summary data were drawn from a GWAS meta-analysis of 175,226 participants of European ancestry from UK Biobank and the Swedish TwinGene cohort.^[21]^ The frailty index was constructed from 44 to 49 self-reported health deficits, including symptoms, disabilities, and physician-diagnosed conditions, accumulated across the life course.

Summary statistics for BMI, HTN, CAD, and T2D were retrieved from the IEU OpenGWAS database.^[22]^ A synopsis of outcome and mediator GWAS sources, sample sizes, ancestry, and phenotype units is presented in Table 1.

**Table 1:** Basic information about the GWAS datasets for the outcome and mediators.

Trait	GWAS-ID	Year	Population	Number of SNPs	Sample size
Frailty index	ebi-a-GCST90020053	2021	European	7,589,717	175,226
Body mass index	ebi-a-GCST006368	2018	European	27,854,527	315,347
Hypertension	finn-b-I9_HYPTENSESS	2021	European	16,380,443	ncase: 42,857 ncontrol: 162,837
Coronary artery disease	ebi-a-GCST005195	2017	European	7,934,254	ncase: 122,733 ncontrol: 424,528
Type 2 diabetes	ebi-a-GCST90018926	2021	European	24,167,560	ncase: 38,841 ncontrol: 451,248

GWAS = genome-wide association study, SNP = single nucleotide polymorphism.

We also reviewed cohort-level information from the original GWAS supplementary files to assess potential sample overlap. The frailty GWAS included 164,610 UK Biobank participants and 10,616 TwinGene participants, whereas the LST GWAS included 448,902 European-ancestry UK Biobank participants with available LST phenotype data. The maximum possible UK Biobank overlap between the exposure and outcome GWAS was therefore estimated at 164,610 participants, corresponding to up to 93.9% of the frailty GWAS sample and 36.7% of the UK Biobank subset within the LST GWAS. This maximum overlap also represented approximately 31.3% of the total LST GWAS sample. These figures should be regarded as upper-bound estimates rather than the exact participant-level overlap, because individual-level identifiers were unavailable in the summary-level data.

### 2.3. Selection of instrumental variables

Independent single nucleotide polymorphisms (SNPs) for the exposure were selected using 3 prespecified criteria. First, variants associated with LST at genome-wide significance (*P* < 5 × 10^−8^) were eligible. Second, to ensure independence, we performed linkage disequilibrium clumping within a ±1000 kb window, retaining the lead SNP and removing correlated variants (*r*^2^ ≥ 0.001). Third, instrument strength was evaluated using the *F*-statistic, and SNPs with *F* > 10 were considered sufficiently strong to limit weak-instrument bias. To reduce potential violations of the independence and exclusion-restriction assumptions, candidate instruments were screened using LDtrait, and variants linked to traits that could plausibly confound the LST-frailty relationship or reflect horizontal pleiotropy were excluded.^[23]^ These traits included educational attainment, BMI, intelligence, and physical activity.

For the first step of the two-step MR mediation analysis, the LDtrait-screened LST instrument pool was used as the starting set for estimating the effects of LST on BMI, HTN, CAD, and T2D. After harmonization with each mediator GWAS, the final number of available SNPs differed across mediator-specific analyses because some variants were unavailable or allele-incompatible. The 7 variants identified by radial MR in the LST-frailty analysis were not automatically excluded from the LST-mediator analyses, because radial MR outliers are outcome-specific. The outlier-corrected LST-frailty estimate was used as the total effect when calculating mediation proportions.

### 2.4. Statistical analysis

We applied 4 MR estimators: IVW, MR-Egger, weighted median, and weighted mode. Multiplicative random-effects IVW was prespecified as the primary estimator because it provides an efficient estimate when the average pleiotropic effect is balanced around zero and can accommodate between-variant heterogeneity by incorporating overdispersion into the standard error.^[24]^ When heterogeneity was present, the IVW estimate was therefore interpreted alongside pleiotropy-robust sensitivity estimators and outlier-detection procedures. The weighted median estimator was used as a complementary method because it can provide a consistent estimate when at least 50% of the total instrument weight comes from valid instruments.^[25]^ The weighted mode estimator was also applied, as it assumes that the largest cluster of instruments with similar causal estimates represents the valid instrument group.^[26]^

A two-step MR framework was used to evaluate potential mediation by cardiometabolic traits. β_1_ denoted the total effect of genetically proxied LST on frailty estimated in the primary MR analysis, β_2_ denoted the effect of LST on each cardiometabolic mediator, and β_3_ denoted the effect of each mediator on frailty. The mediated effect was calculated as β_2_ × β_3_, and the proportion mediated was calculated as (β_2_ × β_3_)/β_1_. All beta estimates are presented on the log-odds scale. Mediation proportions were estimated separately for each mediator and should not be summed because BMI, HTN, CAD, and T2D are biologically and statistically correlated.

Sensitivity analyses were performed to assess robustness. Horizontal pleiotropy was evaluated using the MR-Egger intercept, and heterogeneity was assessed with Cochran *Q*.^[27,28]^ When heterogeneity or pleiotropy was suggested, radial MR and MR-PRESSO were used to identify influential outliers^[29,30]^; IVW estimates were then recalculated after outlier removal, with heterogeneity and pleiotropy reassessed. Leave-one-out analyses were used to examine the influence of each individual SNP on the overall estimates.

## 3. Results

### 3.1. Selection of instrumental variables

Using the prespecified criteria, we identified 115 genome-wide significant SNPs for LST (Table S1, Supplemental Digital Content 1). After LDtrait screening, 17 SNPs associated with potential confounders were excluded (Table S2, Supplemental Digital Content 2). The instruments retained in the outlier-corrected LST–frailty analysis are listed in Table S4, Supplemental Digital Content 3.

### 3.2. MR results of leisure screen time on frailty

**Figure 2.**
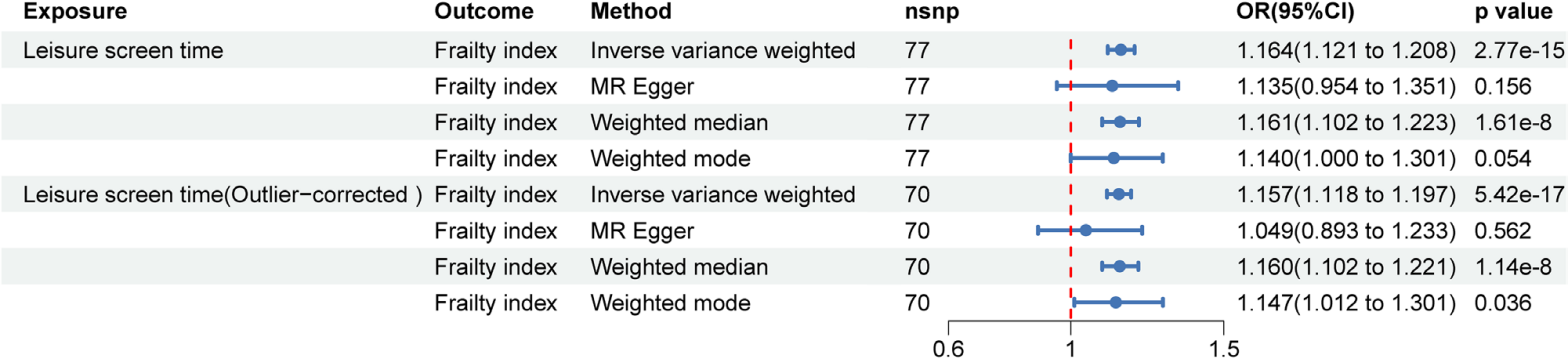
Causal relationships between leisure screen time and frailty.

Two-sample MR indicated that higher genetically proxied LST was associated with greater frailty risk (IVW: odds ratio [OR] = 1.164, 95% confidence interval [CI]: 1.121–1.208, *P* = 2.77e^−15^). Estimates from the MR-Egger, weighted median, and weighted mode methods were directionally concordant (Fig. 2; Fig. S1, Supplemental Digital Content 4; Table 2; Table S5, Supplemental Digital Content 5).

**Table 2:** Main MR results.

Exposure	Number of SNP	Method	OR (95% CI)	*P* value	MR-Egger regression		MR-presso		Cochran *Q* test *P* value
					Egger intercept	*P* value	RSSobs	Global test *P* value	
Leisure screen time	77	Inverse variance weighted	1.164 (1.121, 1.208)	2.77e^−15^	0.000655	.777	103.72	.0457	.0245
		MR Egger	1.135 (0.954, 1.351)	0.156					
		Weighted median	1.161 (1.102, 1.223)	1.61e^−8^					
		Weighted mode	1.140 (0.999, 1.301)	0.054					
Leisure screen time (outlier-corrected)	70	Inverse variance weighted	1.157 (1.118, 1.197)	5.42e^−17^	0.00260	.229	67.65	.652	.607
		MR Egger	1.049 (0.893, 1.233)	0.562					
		Weighted median	1.160 (1.102, 1.221)	1.14e^−8^					
		Weighted mode	1.147 (1.012, 1.301)	0.036					

CI = confidence interval, MR = Mendelian randomization, OR = odds ratio, SNP = single nucleotide polymorphism.

Sensitivity analyses supported the robustness of this association. The MR-Egger intercept provided no evidence of horizontal pleiotropy (*P* = .777), whereas Cochran *Q* indicated heterogeneity (*P* = .025). Although the MR-PRESSO global test was significant (*P* = .046), MR-PRESSO did not identify specific outliers (Table 2). Radial MR subsequently identified 7 outlying variants (Table S3, Supplemental Digital Content 6). After these variants were removed, the LST–frailty association persisted (IVW: OR = 1.157, 95% CI: 1.118–1.197, *P* = 5.42e^−17^). After exclusion, there was no evidence of residual heterogeneity or pleiotropy by Cochran *Q*, the MR-Egger intercept, or MR-PRESSO (Fig. 2; Fig. S1, Supplemental Digital Content 4; Table 2; Table S5, Supplemental Digital Content 5). The outlier-corrected IVW estimate was therefore used as the main result for interpretation. Leave-one-out analyses further indicated that no single SNP unduly influenced the results (Fig. S2, Supplemental Digital Content 7).

### 3.3. MR results of two-step MR analysis

Genetic predisposition to higher LST showed positive causal associations with BMI (IVW: OR = 1.114, 95% CI: 1.069–1.160, *P* = 1.90 e^−7^), HTN (IVW: OR = 1.249, 95% CI: 1.105–1.412, *P* = 3.64 e^−4^), CAD (IVW: OR = 1.164, 95% CI: 1.063–1.273, *P* = .001), and T2D (IVW: OR = 1.283, 95% CI: 1.153–1.427, *P* = 4.35 e^−6^) (Table S6, Supplemental Digital Content 8). Although heterogeneity was observed in some models, there was no evidence of horizontal pleiotropy, supporting the robustness of these findings.

When frailty was modeled as the outcome and each cardiometabolic trait as the exposure, we likewise observed positive associations for BMI (IVW: OR = 1.166, 95% CI: 1.119–1.216, *P* = 5.57 e^−13^), HTN (IVW: OR = 1.087, 95% CI: 1.068–1.106, *P* = 3.81 e^−21^), CAD (IVW: OR = 1.072, 95% CI: 1.050–1.094, *P* = 6.50 e^−11^), and T2D (IVW: OR = 1.047, 95% CI: 1.033–1.060, *P* = 2.07 e^−12^) (Table S6, Supplemental Digital Content 8). Despite some heterogeneity, tests did not indicate horizontal pleiotropy, reinforcing the validity of the results.

In two-step MR analyses, BMI, HTN, CAD, and T2D each showed evidence of partial mediation in the LST-frailty association. The indirect effects (β_2_ × β_3_) were 0.017, 0.019, 0.010, and 0.011, corresponding to 11.4%, 12.7%, 7.2%, and 7.8% of the total effect, respectively (Table 3). These mediation proportions are pathway-specific estimates and should not be summed because the mediators are interrelated.

**Table 3:** Results of the two-step mediation MR analysis.

Exposure	Mediator	Outcome	β_1_ (95% CI)	β_2_ (95% CI)	β_3_ (95% CI)	Mediating effect (95% CI)	Mediating ratio (%)
Leisure screen time	Body mass index	Frailty index	0.146 (0.112, 0.180)	0.108 (0.067, 0.148)	0.154 (0.112, 0.196)	0.017 (0.009, 0.024)	11.4%
	Hypertension	Frailty index	0.146 (0.112, 0.180)	0.222 (0.100, 0.345)	0.083 (0.066, 0.101)	0.019 (0.008, 0.030)	12.7%
	Coronary artery disease	Frailty index	0.146 (0.112, 0.180)	0.151 (0.061, 0.242)	0.069 (0.049, 0.090)	0.010 (0.003, 0.018)	7.2%
	Type 2 diabetes	Frailty index	0.146 (0.112, 0.180)	0.249 (0.143, 0.355)	0.046 (0.033, 0.058)	0.011 (0.006, 0.017)	7.8%

β_1_ =total effect; β_2_ = the causal effect of leisure screen time on cardiometabolic factors; β_3_ = the causal effect of cardiometabolic factors on the frailty index; Mediating effect = β_2_ × β_3_. All β estimates are presented on the log-odds scale. Mediation proportions were estimated separately for each mediator and should not be summed because these cardiometabolic traits are biologically and statistically correlated.

CI = confidence interval, MR = Mendelian randomization.

## 4. Discussion

This MR study provides genetic evidence consistent with a potential causal association between higher LST and increased frailty risk. The association remained robust after radial MR outlier correction and showed broadly consistent directions across complementary MR estimators. In this context, the assumptions underlying the weighted median and weighted mode estimators were considered reasonably plausible because SNPs associated with potential confounders were excluded through LDtrait screening, influential variants were evaluated using MR-PRESSO and radial MR, and no evidence of residual heterogeneity or directional pleiotropy was observed after outlier correction. Nevertheless, these assumptions cannot be empirically proven, and residual pleiotropy remains possible. In two-step MR analyses, BMI, HTN, CAD, and T2D showed separate, modest mediation signals, suggesting that cardiometabolic traits may represent plausible intermediate pathways between screen-based sedentary behavior and frailty.

Our findings align with prior observational studies reporting positive associations between sedentary time and frailty in older adults.^[6,10,31,32]^ However, observational designs cannot fully exclude confounding by socioeconomic status, mental health, comorbidities, or reverse causation. MR strengthens causal inference by using genetic instruments, but its validity depends on instrumental-variable assumptions and does not, by itself, prove that reducing screen time will lower frailty risk in individual patients.

The mediation findings are also consistent with previous evidence linking sedentary behavior to cardiometabolic conditions such as obesity, T2D, and cardiovascular disease, which are established correlates of frailty.^[7–9,33,34]^ Importantly, the estimated mediation proportions were modest and were calculated separately for each mediator. Because BMI, HTN, CAD, and T2D are correlated, these proportions should not be added to infer the total mediated proportion. Although the association was statistically robust, the magnitude of the total effect should be interpreted cautiously. The outlier-corrected IVW estimate corresponded to a modest relative increase in frailty risk, and the mediation proportions for individual cardiometabolic traits were also modest. Because only summary-level GWAS data were available and because baseline frailty prevalence and individual-level exposure distributions were not available in the MR framework, we could not reliably translate these estimates into absolute risk differences or clinically individualized risk increments. Therefore, the findings should be interpreted as evidence supporting a population-level etiologic association rather than as a direct estimate of the absolute benefit expected from reducing screen time in clinical practice.

Several physiological mechanisms may plausibly underlie the observed association. Prolonged LST may displace habitual physical activity, thereby reducing mechanical loading and contributing to loss of muscle mass, lower muscle strength, and functional decline, all of which are central to frailty development.^[35,36]^ Sedentary behavior is also linked to impaired glucose handling and insulin resistance, which may promote obesity and T2D and further amplify low-grade inflammation.^[35,37]^ Inflammation is a recognized feature of frailty, and higher levels of inflammatory markers such as C-reactive protein and interleukin-6 have been associated with sedentary behavior and adverse cardiometabolic states.^[38–40]^ In addition, excessive screen time may contribute to positive energy balance through reduced energy expenditure and increased snack-related caloric intake, thereby increasing obesity-related mechanical and metabolic burden.^[41–44]^ Oxidative stress may provide another convergent pathway, as physical inactivity and cardiometabolic disorders are closely linked to oxidative injury, vascular dysfunction, biological aging, and frailty progression.^[45–47]^ Sleep disruption may represent another unmeasured pathway, as prolonged or evening screen exposure has been associated with shorter sleep duration, delayed sleep timing, and poorer sleep quality,^[48,49]^ which are closely linked to cardiometabolic dysfunction and may contribute to impaired physical function, a core component of frailty.^[50]^ These explanations should be interpreted as biologically plausible pathways rather than direct mechanistic evidence from the present MR analysis.

Several limitations should be considered. First, LST is a heterogeneous, self-reported exposure that may include television viewing, computer use, video gaming, and other screen-based leisure activities. Genetic proxies for LST may therefore capture broader behavioral, cognitive, socioeconomic, or lifestyle-related predispositions rather than screen exposure alone. Although LDtrait screening, MR-Egger, MR-PRESSO, and radial MR were used to reduce and evaluate pleiotropy, residual horizontal pleiotropy cannot be fully excluded. Second, potential sample overlap between the LST and frailty GWAS datasets should be considered. Based on cohort-level information, the maximum possible overlap through UK Biobank was estimated at 164,610 participants, corresponding to up to 93.9% of the frailty GWAS sample and 36.7% of the UK Biobank subset within the LST GWAS. This maximum overlap also represented approximately 31.3% of the total LST GWAS sample. These figures should be interpreted as upper-bound estimates rather than exact participant-level overlap because individual-level identifiers were unavailable. Sample overlap can bias two-sample MR estimates, particularly when instruments are weak. However, all retained instruments had F-statistics greater than 10, reducing the likelihood of substantial weak-instrument bias due to sample overlap, although this concern cannot be completely eliminated. Third, the genetic instruments were derived primarily from individuals of European ancestry, which may limit generalizability to other populations.

Future studies with refined exposure phenotyping, individual-level data, longitudinal follow-up, and interventional evidence are needed to determine whether reducing screen-based sedentary behavior can modify frailty trajectories. Additional analyses incorporating sleep-related traits, physical activity patterns, and detailed cardiometabolic profiles may further clarify the pathways linking LST to frailty.

## 5. Conclusion

This two-sample MR study provides genetic evidence consistent with a potential causal association between higher LST and increased frailty risk. BMI, hypertension, CAD, and type 2 diabetes may each partly mediate this association, but the mediation proportions were modest and should not be interpreted as additive. Further studies using refined exposure phenotyping, individual-level data, and interventional designs are warranted to determine whether reducing screen-based sedentary behavior can help prevent frailty.

## Acknowledgments

We sincerely thank the investigators and participants of the UK Biobank, TwinGene, IEU OpenGWAS, FinnGen, and other contributing consortia and studies for making the GWAS summary statistics publicly available.

## Author contributions

**Conceptualization:** Ye Cao.

**Formal analysis:** Ye Cao.

**Investigation:** Ye Cao, Qunxiong Fan.

**Methodology:** Ye Cao, Qunxiong Fan, Yi Feng.

**Software:** Nan Xia.

**Supervision:** Qing Hao, Yi Feng.

**Validation:** Qing Hao, Yi Feng.

**Visualization:** Nan Xia.

**Writing – original draft:** Ye Cao, Qunxiong Fan.

**Writing – review & editing:** Qing Hao, Yi Feng.
















