# Supplementary figures and images for: Leisure screen time, cardiometabolic pathways, and frailty: A two-step Mendelian randomization analysis
Source: Medicine (Baltimore). 2026 Jul 3;105(27):e49560. doi: 10.1097/MD.0000000000049560 (PMC13336955; doi:10.1097/MD.0000000000049560)

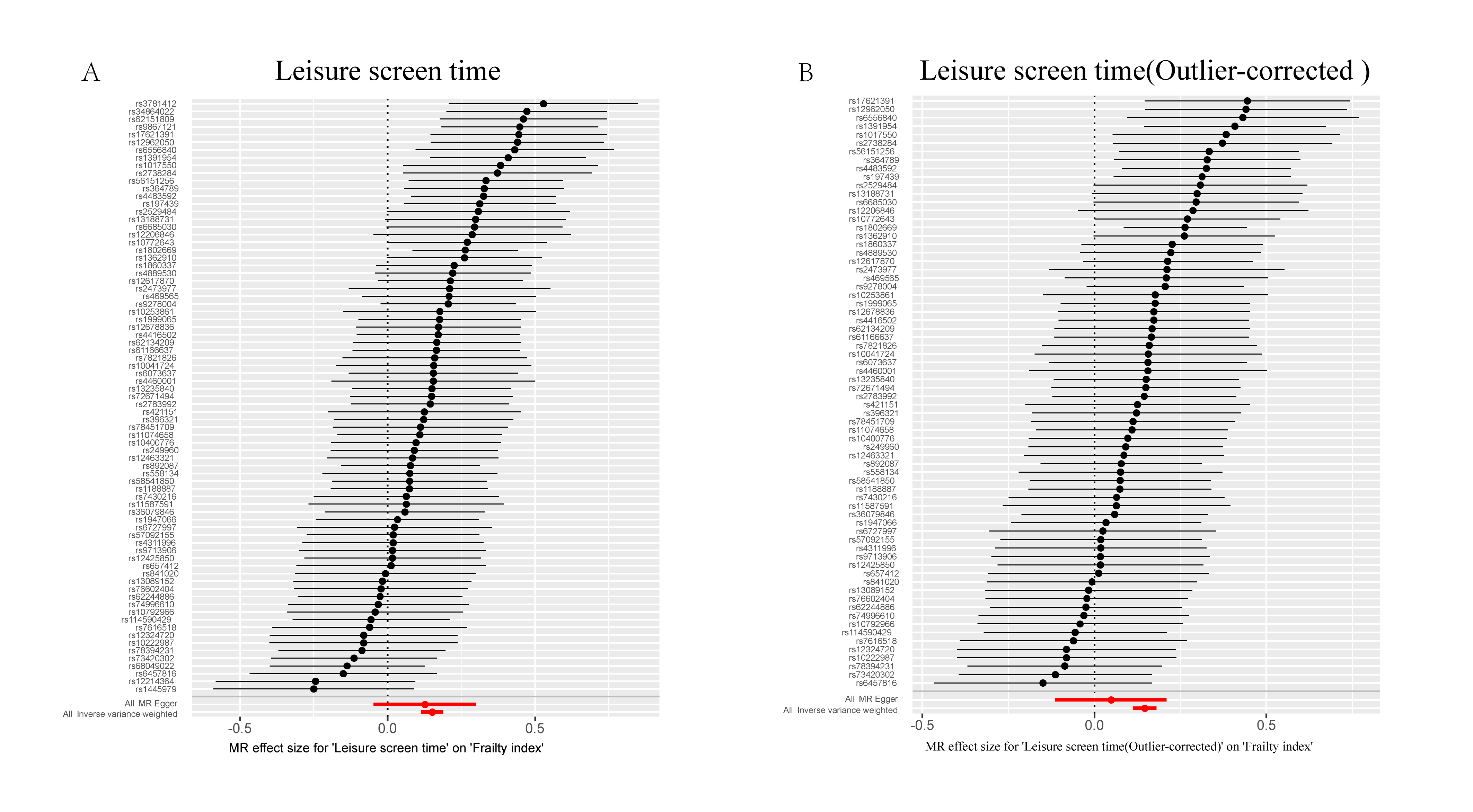

Supplement: Supplementary file 4 [file medi-105-e49560-s004.tif]

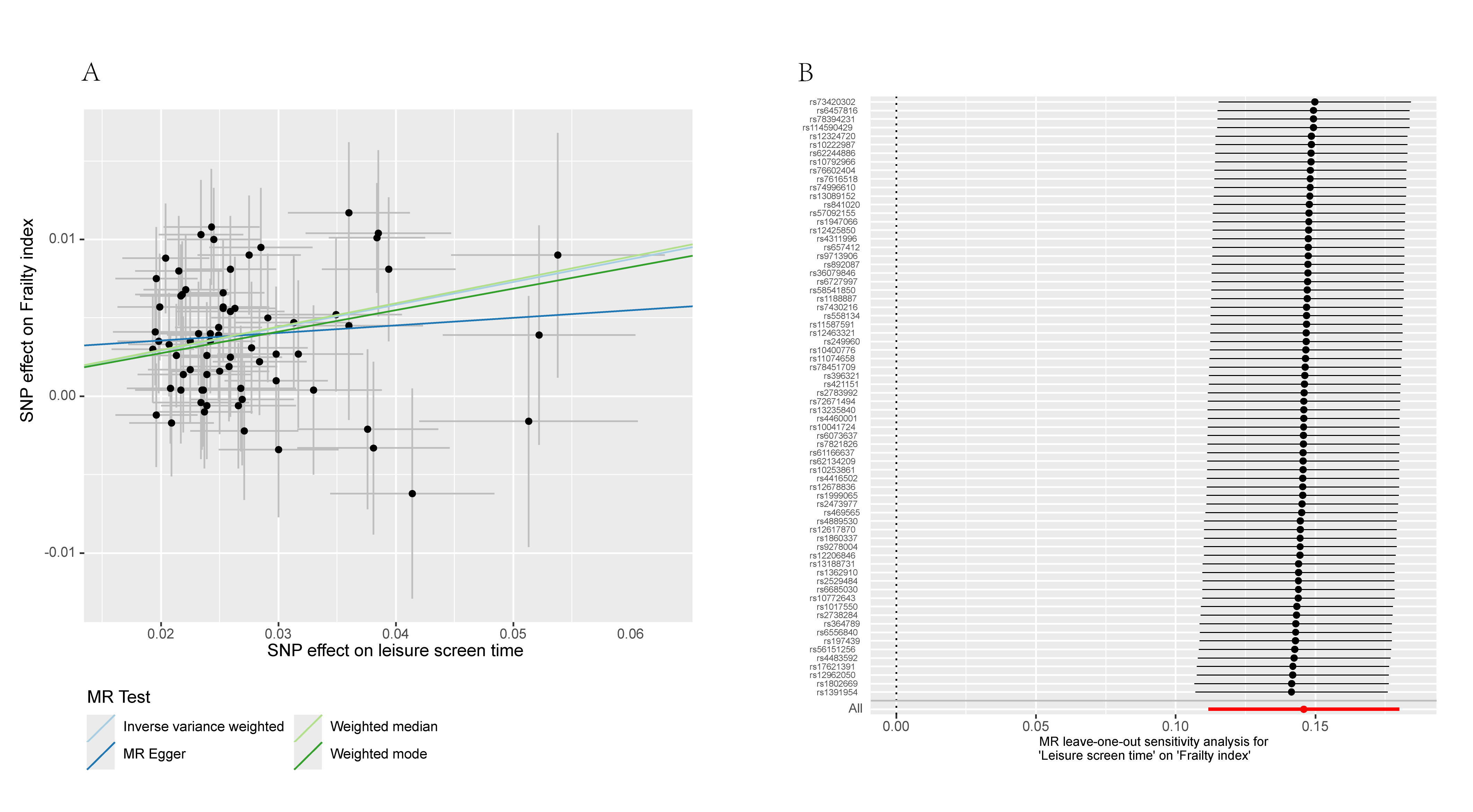

Supplement: Supplementary file 7 [file medi-105-e49560-s007.tif]
